# Cognitive therapy for depression in tuberculosis treatment: protocol for process evaluation of a multicenter hybrid type 1 effectiveness implementation trial in Pakistan

**DOI:** 10.1186/s13063-025-09284-w

**Published:** 2025-12-29

**Authors:** Fayaz Ahmad, Maryiam Rahim, Fatima Khalid Qazi, Zohaib Khan, Shaista Rasool, Zeeshan Kibria, Noor Sanauddin, Mirrat Gul, Farooq Naeem, Muhammad Firaz, Haroon Latif Khan, Saima Sheikh, Saeed Farooq

**Affiliations:** 1 Institute of Public Health & Social Sciences, Khyber Medical University, Peshawar, Pakistan; 2 Office of Research, Innovation & Commercialization, Khyber Medical University, Peshawar, Pakistan; 3 Department of Sociology, University of Peshawar, Peshawar, Pakistan; 4 Mayo Clinic College of Medicine Learning Resource Center, Mayo Clinic College of Medicine and Science, Lahore, Pakistan; 5 University of Toronto, Centre for Addiction & Mental Health, Toronto, Canada; 6 Institute of Mental Health and Behaviour Sciences, Khyber Medical University , Peshawar, Pakistan; 7 School of Medicine, Keele University, Staffordshire, UK; 8 Midlands Partnership NHS Foundation Trust, Stafford, UK

**Keywords:** Process evaluation, Implementation outcomes, Mixed methods, Scale-up

## Abstract

**Background:**

Tuberculosis (TB) remains a significant public health challenge, particularly in low- and middle-income countries (LMICs) such as Pakistan, where TB and depression frequently co-occur, negatively impacting treatment adherence and outcomes. The cognitive therapy for depression in tuberculosis (CONTROL) trial evaluates the effectiveness of a cognitive behavioral therapy (CBT)-based intervention integrated into TB care. Following the Proctor’s framework, this process evaluation aims to assess key implementation outcomes of the trial, including acceptability, adoption, feasibility, appropriateness, fidelity, penetration, and sustainability, to inform the potential scale-up of the intervention within Pakistan’s routine TB care.

**Methods:**

This mixed-methods process evaluation is embedded within the CONTROL randomized controlled trial conducted in Khyber Pakhtunkhwa, Pakistan. Quantitative data collection will include structured implementation outcome measures such as Intervention Appropriateness Measure (IAM), Acceptability of Intervention Measure (AIM), Feasibility of Intervention Measure (FIM), Applied Mental Health Research (AMHR) group tool, Revised Cognitive Therapy Scale (CTS-R), CBT-content delivery assessment checklist, Client Service Receipt Inventory (CSRI), and trial administrative data logs. Qualitative data will comprise semi-structured interviews and focus group discussions. Data collection will be at three points: 8-, 24-, and 32-week post-randomization across 12 TB care facilities. Quantitative data will be analyzed descriptively, while qualitative data will be analyzed thematically, followed by triangulation of findings.

**Discussion:**

The process evaluation will inform the implementation of CBT-based intervention within TB care. It will also identify barriers and facilitators for integrating mental health care in the TB control program for future scale-up. Findings will inform policymakers on the feasibility of incorporating mental health interventions into routine TB care, support improved patient outcomes, and contribute to global implementation science on integrated mental health care for chronic diseases.

**Trial registration:**

ISRCTN10761003. Registered on November 2023.

## Introduction

Tuberculosis (TB) continues to be one of the leading causes of death worldwide, posing a significant public health challenge. Despite advancements in diagnostics, treatment protocols, and global TB control strategies, the disease remains a burden, especially in low- and middle-income countries (LMICs). According to the World Health Organization (WHO) 2024 report, approximately 10.8 million people contracted TB in 2023, with 1.25 million deaths attributed to the disease [1]. Over 95% of these cases and deaths occur in LMICs, where health systems often struggle to provide consistent care due to resource constraints, socio-political challenges, and competing healthcare priorities [2].

Pakistan ranks among the top five high-burden countries for TB, contributing significantly to global morbidity and mortality [2]. The country also has one of the highest rates of multidrug-resistant TB (MDR-TB), further complicating TB control efforts. Annually, Pakistan reports over 500,000 new TB cases [2], with a substantial proportion facing treatment interruptions due to socioeconomic barriers, stigma, and limited access to care. TB imposes profound economic, social, and psychological impacts on affected individuals and their families, often exacerbating the cycle of poverty and illness [3].

Beyond its direct physical health implications, TB frequently co-occurs with mental health disorders, particularly depression and anxiety [4]. These psychological conditions are prevalent among TB patients, with studies estimating that up to 50% of patients experience depressive symptoms during their treatment [5]. Poor mental health can negatively impact TB outcomes through various pathways related to the disease. This bidirectional relationship between TB and mental health creates a complex interplay: the physical and social burdens of TB can trigger or exacerbate mental health issues, while depression and anxiety impair adherence to anti-TB treatment (ATT), increasing the risk of treatment failure and worsening clinical outcomes [6].

Despite ample evidence, mental health care remains a neglected component of TB control programs globally, including in Pakistan [7]. TB control efforts primarily focus on detecting and treating the infection, often overlooking the psychological needs of patients [8]. This gap in care highlights an urgent need for integrated approaches that address both the physical and mental health aspects of TB. Such integration can improve patient outcomes and align with global health priorities, emphasizing holistic, patient-centered care models [9].

Integrating mental health interventions into routine TB care has shown promise in improving both TB and mental health outcomes [7]. Cognitive behavioral therapy (CBT), an evidence-based approach to managing depression and anxiety, has demonstrated effectiveness in reducing depressive symptoms and enhancing adherence to medical treatment [10, 11]. In LMICs, task-sharing approaches—where non-specialist health workers are trained to deliver these interventions—have emerged as a practical and scalable solution [12]. These approaches leverage existing healthcare infrastructure and address workforce limitations by enabling lay health workers to provide mental health care under supervision [13]. Task-sharing has been successfully implemented in other disease contexts, showing potential for adaptation in TB care programs [14].

### A brief overview of the trial

The COgNitive Therapy for depRessiOn in tubercuLosis treatment (CONTROL) trial aims to address the dual burden of TB and depression in Pakistan [15]. It is a pragmatic parallel arm (intervention and control arm), randomized controlled trial (RCT) conducted across TB treatment centers in Khyber Pakhtunkhwa (KP), Pakistan, evaluating the clinical and cost-effectiveness of CONTROL intervention in a randomised controlled trial [15]. In these TB centers, the program is implemented through the Directly Observed Therapy Short Course (DOTS) strategy, ensuring structured service delivery to patients. The CONTROL CBT-based intervention aims to improve the outcomes both for depression and TB in people with TB and MDR-TB. The trial tests its effectiveness and cost-effectiveness and examines how the intervention can be implemented in the real-world setting of the Provincial TB Control Programme, KP (PTP-KP) in Pakistan [15]. Patients in the intervention arm receive both Enhanced Treatment as Usual (ETAU) and the CBT intervention, whereas patients in the control arm receive only ETAU. The ETAU treatment includes the Mental Health Gap Action Programme (mhGAP). In this model, TB health workers (TBHWs)—non-specialist healthcare providers already engaged in TB care and also called TB DOTS facilitators—are trained to deliver CBT sessions to TB patients diagnosed with depression. This approach allows for the efficient use of available resources and ensures that patients can access mental health care within their existing care pathways. Participants in the CONTROL intervention group will undergo six sessions of a CBT-based intervention; the content and modes of intervention were adapted based on the Southampton framework used previously in Pakistan [16]. The Southampton Adaptation Framework is utilized to culturally adapt CBT by considering a patient’s cultural background and religious affiliation, thereby enhancing mental health outcomes [17]. This framework concentrates on three main areas: awareness of culture and religion, assessment and engagement with cultural issues, and modifications in therapy to align with local values and norms [17]. CBT therapy aims at addressing depression while also employing problem-solving and motivational techniques to improve compliance with ATT. Each therapy session will be conducted one-on-one, lasting between 40 and 60 min [15]. The design and methodology of the CONTROL trial are detailed in its published protocol [15], while this paper focuses on the methodology used for the process evaluation of the CONTROL trial.

While RCTs are the gold standard for evaluating clinical outcomes and cost-effectiveness, they often fail to capture the underlying mechanisms determining intervention success or failure [18]. RCTs typically focus on clinical outcomes such as morbidity and intervention effectiveness, neglecting the contextual factors, implementation processes, and stakeholder experiences that influence these results [19]. The requirements to publish the methodology and protocols for the randomized controlled trials have helped the reporting and quality of RCTs over the last two decades. The same is not valid for the process evaluation. The process evaluation in clinical trials informs the key stages in implementing trial procedures and how these are implemented in the real world, which is rarely captured in the clinical trials reporting.

The limited reporting of trial processes and implementation in real-world settings reduces the generalizability and scalability of trial findings, particularly in complex health systems [20]. The UK Medical Research Council’s (MRC) framework for designing and evaluating complex interventions (programs) recommends process evaluations be undertaken as part of all randomized trials, as they describe important contextual factors and can clarify causal mechanisms to aid the interpretation of trial findings and explain variations in trial outcomes [20]. Process evaluations address this gap by systematically investigating how interventions are delivered, received, and sustained in real-world settings [21]. They provide insights into the “how” and “why” behind trial outcomes [22], enabling policymakers and practitioners to adapt and scale up successful interventions. Process evaluations are especially critical in hybrid trials like CONTROL, where effectiveness and implementation are key research components [20]. By measuring these outcomes, we can identify mechanisms and causal relationships in implementation processes, ultimately strengthening the evidence for effective implementation [23]. In this context, Proctor’s framework of implementation outcomes will guide the process evaluation of the CONTROL trial. This framework identifies eight key dimensions essential for understanding complex interventions’ implementation: acceptability, adoption, appropriateness, feasibility, fidelity, implementation cost, penetration, and sustainability [23].

The findings from the process evaluation will generate evidence to inform CONTROL’s adoption and scale-up within Pakistan’s TB control program by exploring the intervention’s contextual and operational dynamics. Moreover, the insights will contribute to the broader evidence on integrating mental health care into chronic disease management in LMICs, addressing a critical gap in global health research. This paper is a protocol for a trial’s process evaluation, aiming to capture the multifaceted implementation aspects and identify actionable strategies for the scale-up of the intervention. The specific objectives of the process evaluation of the CONTROL trial are to:

1. Describe the acceptability, appropriateness, adoption, and feasibility of the CONTROL study and CBT intervention within the routine TB control program of KP Pakistan.
2. Describe the fidelity to CONTROL CBT intervention delivery.
3. Describe the penetration, sustainability, and implementation costs of the mental health assessment measures and CBT therapy within the routine TB control program of KP Pakistan.
4. Explore the barriers and facilitators to the sustainability and scale-up of CBT therapy within KP Pakistan's routine TB control program.

## Methods

This protocol provides a detailed methodology for the comprehensive process evaluation of the CONTROL study. Employing quantitative and qualitative methods, the evaluation will examine the practical challenges, contextual alignment, and effectiveness of the intervention within a TB care setting [24].

### Study design and settings

Through a concurrent mixed-method approach using quantitative and qualitative methods, we will comprehensively evaluate key implementation outcomes defined by Proctor’s framework (Table 2) [23]. The CONTROL CBT intervention will be delivered over nine months at 12 purposively selected TB care facilities (Study centers) at two study sites, districts Peshawar and Haripur, located in Khyber Pakhtunkhwa, Pakistan [15]. The selected facilities represent a mix of primary, secondary, and tertiary care settings, ensuring the intervention’s applicability and evaluation across various healthcare system levels.

Although extensively trained before the pilot phase of the trial, the TBHWs will undergo a “ten-day training course” designed to help them understand the core principles of CBT, acquire practical techniques for managing depression and anxiety in TB, and enhance their communication skills. In these trainings, master trainers, initially trained by a team comprising senior psychiatrists and a psychologist with extensive expertise in CBT delivery, will subsequently train the TBHWs to implement the intervention [15]. Medical officers (MOs) responsible for treating TB patients at each facility will receive training in the Mental Health Gap Action Programme (mhGAP). The MOs will deliver mhGAP, as enhanced treatment as usual (ETAU), to both arms of TB patients. The mhGAP training for the MOs will be delivered by a psychiatrist. This training will be based on the World Health Organization’s mhGAP intervention guide [25], ensuring alignment with standardized global mental health guidelines [15]. After each training session, participant feedback will be systematically collected to evaluate satisfaction levels. A feedback form, translated into Urdu for cultural and linguistic appropriateness, will be utilized.

### Data collection

Several quantitative and qualitative approaches will be used to gather insights from TB patients, TBHWs, medical officers, the trial team, and TB program managers. Tables 2 and 3 outline these methods and their respective participants. Table 1, mentioned below, represents the scheduled timing of all data collection tools during the trial. We first explain the details of the quantitative methods for the first three objectives of the study. We then explain the qualitative methods overall and specifically for objective 4 of the study.

**Table 1:**
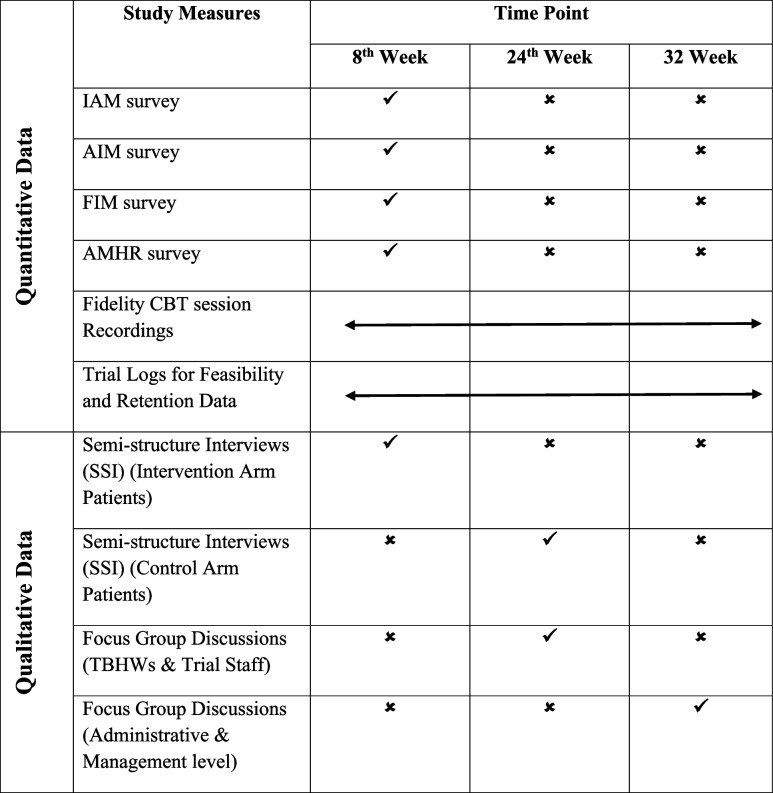
Schedule of the CONTROL intervention process evaluation data collection methods

### Quantitative data

Quantitative data collection for the process evaluation will systematically measure key implementation outcomes related to integrating mental health screening and CBT therapy within the routine TB control program in KP, Pakistan. Data will be collected using routine trial logs (Excel sheets consisting of prescreening, screening, recruitment data, retention data, and CBT records), structured checklists and questionnaires, patient records, and fidelity assessment tools.

## Acceptability, appropriateness, adoption and feasibility

Four quantitative tools will collect data on Proctor et al.’s four implementation outcomes: acceptability, appropriateness, adoption, and feasibility [23].

### Data collection tools

Acceptability, appropriateness, and feasibility of the CONTROL study and CBT intervention will be assessed at 8 weeks post-randomization using three valid and reliable scales developed by Weiner et al., the Acceptability of Intervention Measure (AIM), Intervention Appropriateness Measure (IAM), and the Feasibility of Intervention Measure (FIM) [26]. For sampling, measuring tools, and data collection time points, see Table 2. Each scale consists of four thematic questions with five responses ranging from “completely disagree” to “completely agree” on a five-point Likert scale [27]. Selected for their conceptual distinctiveness, AIM, IAM, and FIM provide valuable insights into the implementation process while also accounting for potential empirical interrelations [27]. They enhance the evaluation by providing robust, evidence-based metrics to assess the feasibility and acceptability of the intervention in complex healthcare settings [27].

**Table 2:** Implementation outcomes for CONTROL trial process evaluation

Quantitative measures					
**Sr. No**	**Outcomes**	**Brief description**	**Measuring tool**	**Data collection timepoint**	**Group (patients, therapist, trial team)**
**1**	**Appropriateness**	Appropriateness refers to the perceived fit, relevance, compatibility, suitability, usefulness, and practicability of the CBT intervention within the TB care settings, as experienced by facilitators and participants	Intervention Appropriateness Measure (IAM)	8-week	10% of randomly selected patients in the intervention arm
			The Applied Mental Health Research Group (AMHR)-appropriateness subscale	8-week	10% of randomly selected patients in the intervention arm
**2**	**Feasibility**	The CBT intervention’s actual fit, utility, and practicability within routine TB care, assessing the extent to which TBHWs can effectively deliver CBT and patients can access and engage with it given the available resources, time, and training	Feasibility of Intervention Measure (FIM)	32-week	All trial staff (DOTS and the trial team)
			The Applied Mental Health Research Group (AMHR)- feasibility subscale	8-week	10% of randomly selected patients in the intervention arm
			% assigned to the intervention who agreed to enroll in the trial	After the definitive trial, all relevant data will be retrieved from the trial team’s data logs	
**3**	**Adoption**	Adoption refers to the early uptake, utilization, and initial implementation of the CBT intervention within The CONTROL Trial and their intention to integrate it into routine TB care	The Applied Mental Health Research Group (AMHR)-adoption subscale	8-week	10% of randomly selected patients in the intervention arm
**4**	**Acceptability**	Acceptability refers to participants' satisfaction and perceptions regarding various aspects of the CBT intervention delivered under The CONTROL Trial, including its content, complexity, ease of participation, delivery method, and perceived credibility	The Applied Mental Health Research Group (AMHR)-acceptability subscale	8-week	10% of randomly selected patients in the intervention arm
			Acceptability of Intervention Measure (AIM)	8-week	10% of randomly selected patients in the intervention arm
			Retention in the number of sessions attended and % of those who dropped out of the intervention	After the definitive trial, all relevant data will be retrieved from the trial team's data logs	
**5**	**Fidelity**	The extent to which the CBT intervention is delivered as intended, ensuring adherence to the structured protocol, maintaining integrity in session delivery, and upholding the quality of program implementation by TBHWs within routine TB care	The Revised Cognitive Therapy Scale (CTS-R)	Ongoing process (first to sixth week post-randomization)	10% of all the sessions, delivered by the DOTS facilitator
			CBT content delivery assessment scale		
**6**	**Penetration**	The extent to which the CBT intervention is institutionalized within the TB care system, assessing its spread across healthcare facilities and the level of service access for TB patients needing psychological support	Data from the routine trial logs	After the definitive trial, all relevant data will be retrieved from the trial team’s data logs	
**7**	**Sustainability**	The extent to which the CBT intervention is maintained, continued, and durably integrated into routine TB care beyond the CONTROL trial, assessing its incorporation into health system structures, institutionalization within policies, and sustained use by TBHWs and patients	Administrative Data logs	After the definitive trial, all relevant data will be retrieved from the trial team’s data logs	
**8**	**Implementation Cost**	The financial investment required to deliver the CBT intervention within routine TB care, considering marginal costs, cost-effectiveness, and cost–benefit concerning its scalability and sustainability	Administrative Data logs	After the definitive trial, all relevant data will be retrieved from the trial team’s data logs	

The Applied Mental Health Research Group (AMHR) developed a comprehensive measure to evaluate multiple implementation domains, encompassing four constructs: acceptability (17 items), appropriateness (13 items), feasibility (14 items), and adoption (8 items). Each construct is assessed using a four-point Likert scale, with response options ranging from 0 “Not at all” to 3 “A lot” [28]. The AMHR tool is a validated instrument explicitly designed to allow adaptation to specific interventions.

We will use the AMHR tool for triangulation and to strengthen the findings on the four implementation outcomes. The AMHR will be culturally adapted to ensure cultural and contextual relevance. This will include the expert validity via online Google Forms. The experts include public health professionals, psychiatrists, CBT specialists, psychologists, and medical education experts. The experts will evaluate each item for relevance, clarity, and importance. Based on the three domains, a Content Validity Index (CVI) will be calculated for their responses to assess and confirm the tool’s appropriateness and cultural adaptability for the target population. It is recommended that a scale demonstrates excellent content validity with I-CVI values of 0.78 or above and S-CVI/Ave values of 0.9 or higher [29–31].

### Data collection and sampling

The above quantitative measures will be applied to collect data from the intervention group. The process evaluation will involve a carefully selected population from various groups, including patients, CBT therapists (TBHWs), and trial staff, to ensure comprehensive representation and validity of findings.

Data collection from all TBHWs and trial staff will utilize FIM. Research assistants from the process evaluation team will facilitate the completion of the tool by TBHWs and trial staff at the 32-week post-randomization mark, ensuring standardized and accurate data collection. The IAM and AIM will assess 10% of patients who have completed therapy sessions. Data collection will occur 8 weeks after randomization by the research assistants. The tools (FIM, IAM, AIM) will be translated into the local language to ensure cultural and linguistic relevance.

The AMHR subscales for appropriateness, adoption, feasibility, and acceptability will be administered to 10% of randomly selected patients in the intervention arm at 8 weeks post-randomization. The AMHR tool will be updated based on the CVI results and subsequently translated into the local language.

Retention rates, encompassing the number of sessions attended and the proportion of participants who discontinued the intervention, will be systematically evaluated [15]. Furthermore, the enrollment rate, defined as the percentage of individuals assigned to the intervention who consented to participate, will also be analyzed. These data will be extracted from the trial logs upon the completion of the definitive trial, offering valuable insights into participant engagement, adherence, and overall intervention feasibility and acceptability [15].

## Fidelity

### Data collection tools

CBT sessions will be audio recorded to evaluate the fidelity to CONTROL CBT intervention delivery using the Revised Cognitive Therapy Scale (CTS-R) and CBT delivery assessment checklist (CDAC). These two tools will comprehensively evaluate fidelity, assessing therapeutic competence and adherence to intervention content (Table 2).

The CTS-R is a well-established instrument for assessing therapists’ competence in delivering CBT [32]. The scale comprises 12 items, evaluating several key aspects ranging from agenda setting to homework setting. Each item is scored on a 7-point Likert scale ranging from 0 to 6, where 0 indicates “the absence of a competence feature or highly inappropriate performance” and 6 indicates “excellent performance, or very good even in the face of patient difficulties” [32]. These items are categorized into three main sections: general interview procedures, interpersonal effectiveness, and specific cognitive-behavioral techniques. This structured approach ensures a comprehensive assessment of both the therapeutic process and the delivery of CBT techniques. The maximum score on the scale is 72 (12 × 6). The Newcastle Cognitive Therapy Center has set a minimum competence standard of 36, an average of 3 marks per item [32].

The second tool that will be used to measure fidelity is the CBT delivery assessment checklist; this will evaluate how the intervention content components were delivered and the degree of adherence. The checklist was developed by CBT master trainers based on the CBT content and was subsequently validated by CBT experts FN and MG. It was piloted and modified for use in the definitive trial. Each item on the checklist is marked on a 5-point Likert scale from 0, “not implemented,” to 4, “fully implemented.”

### Data collection and sampling

The audio recordings of each CBT session will be assessed by two trained independent raters (experts in CBT) to ensure adherence to the intervention protocol using CTS-R and the CBT delivery assessment checklist. A randomization procedure, without replacement [33], is applied to randomly select 10% of the IDs for each CBT session, resulting in 35 participant IDs per CBT session; 210 sessions will be recorded. We will utilize randomization without replacement to ensure unbiased and representative audio selection for fidelity assessment. This method guarantees each session has an equal chance of selection, preventing the overrepresentation of specific facilitators and enhancing fairness across all sessions. Research assistants responsible for recording CBT sessions will undergo structured training to ensure adherence to the standardized protocol for session documentation. Informed consent will be obtained from all participants before recording.

## Penetration, sustainability and implementation costs

### Data collection tool

Penetration and sustainability will be assessed using routine trial logs after the definitive trial, focusing on policymakers’ acceptance of mental health measures and evaluating the sensitivity and specificity of the Patient Health Questionnaire short version (PHQ-2) in routine depression screening within the TB care program. In the CONTROL definitive trial, TBHWs will (pre)screen all patients presenting at the selected TB centers on PHQ-2 and assess the frequency of depressed mood and anhedonia over a 2-week period. Being a simple two-question screening tool, it will be easy to implement in the routine, and could be used by healthcare workers at TB centers [15]. The data will also help explore the potential adaptation of PHQ-2 to screen depression in other chronic infections prevalent in the region, providing robust insights into the implementation and scalability of the study measures across diverse healthcare settings.

Implementation costs are influenced by three main factors: the treatment’s complexity, the implementation strategy’s intricacy, and the setting where the treatment is delivered. Consequently, the total cost of implementation depends on the specific intervention, the chosen strategy, and the delivery environment [23]. Implementation costs will be measured using the Client Service Receipt Inventory (CSRI) [34]. The evaluation will mainly be approached from the standpoint of the health system, including (a) expenses associated with the implementation of the intervention during the trial, (b) utilization of additional healthcare and related services by the participants, and (c) costs incurred by patients and their families.

### Data collection and sampling

Data collection will include routine trial logs to document the number of TB patients screened, further assessed, and those receiving CBT intervention. Screening penetration will be measured by calculating the proportion of TB patients screened using PHQ-2 and their referral rates to mental health services. Usability and sustainability will be assessed by recording the time required to complete PHQ-2 screening, dropout rates, and follow-up adherence among CBT recipients.

The expenses related to the implementation will be evaluated by examining the costs and cost-effectiveness measures used during the trial. This evaluation will consider the expenses of the CONTROL intervention and enhanced treatment as usual (ETAU). An adapted version of the Client Service Receipt Inventory (CSRI) will be utilized to assess costs, similar to our previous methodology for cost-effectiveness analysis of psychological intervention in the region [35]. This assessment will encompass visits to primary care and outpatient services, medication costs, travel time, transportation expenses, and days lost from work. Current prices or unit costs will be obtained from national sources, validated, and, if necessary, extrapolated from UK equivalents to be applied to these resource categories, allowing for an estimate of overall and individual costs.

## Barriers and facilitators to sustainability and scale-up

Qualitative methods will be employed to explore the barriers and facilitators to the sustainability and scale-up of CBT therapy within routine TB control. Focus group discussions (FGDs) and semi-structured interviews (SSIs) will be conducted with key stakeholders, including TB patients who have received CBT, TBHWs involved in delivering the intervention, managers overseeing the TB control program, policymakers, and the CONTROL trial staff. Detailed methods for this objective are explained in the section below.

### Qualitative data

The implementation outcomes in this process evaluation will also be explored through SSIs and FGDs with different groups of participants. Findings from the qualitative data will be triangulated with quantitative findings to present a more authentic and holistic picture of the process.

### Data collection tool

Topic guides will be developed based on Proctor’s model of implementation outcomes [23] to guide qualitative data collection. They will then be reviewed and finalized with input from a qualitative expert, a public health expert, and members of the PPIE advisory group. These guides will serve as a structured framework to capture in-depth insight into participants’ experiences, perceptions of the intervention, the contextual and operational factors influencing its delivery, and barriers and facilitators to scale up. A total of six distinct topic guides will be developed, tailored to intervention arm patients, control arm patients, TBHWs, MOs, trial staff, and health administrators, based on their respective roles in the trial.

### Data collection and sampling

A comprehensive sampling strategy will include a wide range of insights and diverse perspectives targeting key stakeholder groups, providing an in-depth understanding of the implementation process and factors influencing the intervention's scalability. Patients who completed the intervention (attended five or all six sessions) will be sampled to conduct 10–15 SSIs at 8 weeks post-randomization, while patients who partially completed the intervention (attended 1–4 sessions) will contribute 5–10 SSIs in the same timeframe (Table 3). From a policy-level perspective, 8–12 SSIs will be conducted with TB control program managers, health administrators, pulmonology health care workers from private practice, and infectious disease program managers after the final CBT session delivery to all trial intervention arm patients. Moreover, 5–10 SSIs will be conducted with control arm patients after 24 weeks post-randomization to capture their perspectives. We will also conduct seven FGDs after 24 weeks post-randomization with TBHWs (*n* = 2), CONTROL trial staff (*n* = 3), and TB program medical officers (*n* = 2).

**Table 3:** Qualitative data collection using topic guides based on Proctor et al.’s implementation outcomes and exploring barriers and facilitators to future scale-up

Qualitative measures			
**Group of participants**	**SSIs**	**FGDs**	**Timeline**
Patients who completed the intervention (attended five or all six sessions), the maximum variation	10–15	Nil	After 8 weeks post-randomization
Patients who couldn’t complete the intervention (attended 1–4 sessions)	5–10	Nil	After 8 weeks post-randomization
Patients in the control arm	5–10	Nil	After 24 weeks post-randomization
DOTS facilitators		02	After 24 weeks post-randomization
Medical officers		02	After 24 weeks post-randomization
Trial staff		03	After 24 weeks post-randomization
TB control program management, (ii) health administrators, (iii) TB and Pulmonology health care workers diagnosing and treating TB in private practice, and (iv) the managers of the infectious disease program	8–12	Nil	After the delivery of the final session to all the trial participants
Total	30–35	07	

All interviews will be audio-recorded using secure, encrypted digital devices to ensure accurate data capture. Recording will begin only after participants provide explicit verbal and written consent. Sessions will be conducted in private, quiet settings to maintain confidentiality and minimize disruptions. Audio recordings will be securely stored in password-protected files on encrypted devices. Access to these recordings will be restricted to authorized research team members involved in data analysis. Identifiable information will be removed during transcription to ensure participant anonymity, and all data will be securely archived following ethical guidelines.

## Data analysis

### Quantitative data analysis

We will employ descriptive and inferential statistical methods to analyse the CONTROL study and CBT intervention’s implementation outcomes—acceptability, appropriateness, adoption, and feasibility. Data from the AIM, IAM, FIM, and AMHR scales will be analysed to assess central tendencies and variability. Regression analyses will be conducted to explore relationships between implementation outcomes and participant characteristics. Retention and enrolment rates will be calculated as proportions, with chi-square tests to examine group differences.

Two independent reviewers, both experts in CBT, will assess a randomized selection of 10% of the audio-recorded CBT sessions to analyse the fidelity of the CONTROL CBT intervention through CTS-R and a CBT delivery assessment checklist. According to the Newcastle Cognitive Therapy Centre, a minimum competence standard is set at 36, with higher scores indicating greater therapeutic competence. Descriptive statistics will summarize the distribution of scores across sessions, and inter-rater reliability will be assessed using intraclass correlation coefficients to ensure consistency between reviewers. Similarly, the delivery assessment checklist evaluates adherence to the intervention’s content, and for each session, item scores will be aggregated to produce a total adherence score. Descriptive statistics will describe the adherence levels, and inter-rater reliability will be calculated to confirm reviewer consistency.

Penetration analysis will calculate the proportion of TB patients screened with the PHQ-2 and those referred for mental health services. High screening and referral rates will indicate successful penetration of mental health assessments into the TB program. Cost analysis will utilize data from the CSRI to compute the total and per-patient costs of implementing the mental health interventions. This will include direct costs (e.g., training, therapy sessions) and indirect costs (e.g., patient travel, lost productivity). The cost-effectiveness of the intervention will be evaluated by comparing the incremental costs to the health outcomes achieved, such as reductions in depression and anxiety symptoms.

### Qualitative data analysis

The qualitative data collected through SSIs and FGDs will be analyzed using the framework analysis approach, a systematic and flexible method suited to implementation research contexts [36]. This approach comprises a series of structured steps to ensure comprehensive analysis. Initially, researchers will familiarize themselves with the data by thoroughly reviewing audio recordings and transcripts and identifying key concepts and recurrent themes. Subsequently, a thematic framework will be developed, informed by Proctor et al.’s implementation outcomes model and emerging themes, focusing mainly on constructs such as acceptability, feasibility, adoption, and scalability.

Thematic coding will then be applied systematically to the transcripts, with text segments accurately labeled and categorized under the identified themes to maintain consistency and traceability. The data will be organized into a matrix format, aligning key excerpts with thematic categories and stakeholder groups to provide a structured representation. This matrix will facilitate the identification of patterns, relationships, and variations across the data, which will be interpreted within the context of the implementation framework to explore barriers, facilitators, and contextual factors impacting the intervention.

To ensure methodological accuracy, a subset of transcripts will undergo independent coding by two researchers, with any discrepancies resolved through discussion or consultation with a third researcher. Confidentiality protocols will be strictly followed, including anonymizing transcripts to remove all identifiable information. Data will be securely stored on password-protected systems, accessible only to authorized personnel.

## Discussion

Process evaluation is crucial in public health programs, as it examines how interventions are adapted across diverse settings to fit the local context [37]. Therefore, we are conducting a process evaluation of the CONTROL trial to assess the implementation of the CBT intervention within the real-world TB program. Overseeing a high-quality process evaluation of a trial necessitates actively involving all stakeholders in the intervention’s development and implementation phases [20]. In line with this, our study will involve key stakeholders to ensure the intervention is contextually relevant and well-informed. We will seek expert opinions on the tools and PPIE members who will contribute to finalizing the qualitative topic guides. Moreover, we will incorporate feedback from TB patients, TBHWs, trial staff, and policymakers at every trial stage to capture their perspectives and enhance the intervention’s acceptability and feasibility.

For process evaluation of complex interventions, the evaluator must maintain sufficient independence to observe all aspects of the trial and stakeholder activities objectively, ensuring transparent and unbiased reporting [20]. In our study, we will uphold this principle by keeping the process evaluation team separate from the trial team, preventing potential contamination between the two. This separation will safeguard the integrity of both processes, allowing for impartial data collection and minimizing any influence on trial outcomes. Maintaining distinct roles will ensure that our evaluation accurately captures the implementation without interfering with the trial’s core objectives.

The study presents several strengths that contribute to its robustness. It will integrate quantitative and qualitative methods, guided by Proctor’s implementation outcomes model, ensuring an organized approach to process evaluation. Moreover, the cultural relevance of the study will be reinforced by adapting and validating data collection tools for the local context, including translations into Urdu and Pashto. The robust training model, utilizing a cascade approach, will support the scalability and sustainability of the intervention, capitalizing on existing resources within the TB program. However, the study also has several limitations. Its findings may be limited in generalizability as they focus on specific districts with unique demographic and healthcare dynamics. The study’s resource intensity, involving extensive training, multiple data collection tools, and independent reviews, may be challenging to replicate in resource-limited settings. Lastly, participant attrition, such as dropouts among TBHWs or patients, can affect completeness and fidelity assessments.

### Trial status

Protocol Version 1.0, 17th March 2025. Currently, the trial is ongoing. Recruitment for the process evaluation will begin from 10th April 2025 and is expected to be completed by 31st January 2026.

## Data Availability

Not applicable.

## Abbreviations

TB: Tuberculosis
LMICs: Low- and middle-income countries
CONTROL: COgNitive Therapy for depRessiOn in tubercuLosis treatment
CBT: Cognitive behavioral therapy
ATT: Anti-TB treatment
RCT: Randomized controlled trial
MDR-TB: Multidrug-resistant tuberculosis
PTP: Provincial TB Control Programme
DOTS: Directly Observed Therapy, Short-Course
TBHW: TB Health Worker (DOTS Facilitator)
MO: Medical officer
mhGAP: Mental Health Gap Action Programme
IAM: Intervention appropriateness measure
AIM: Acceptability of intervention measure
FIM: Feasibility of Intervention Measure
AMHR: Applied Mental Health Research Group
CTS-R: Revised Cognitive Therapy Scale
CSRI: Client Service Receipt Inventory
PPIE: Patient and Public Involvement and Engagement
MRC: Medical Research Council
WHO: World Health Organization
SSI: Semi-structured interviews
FGDs: Focus group discussions
PHQ-2: Patient Health Questionnaire-2
